# Genome Sequence of Highly Virulent *Pseudomonas aeruginosa* Strain VA-134, Isolated from a Burn Patient

**DOI:** 10.1128/genomeA.01662-15

**Published:** 2016-01-28

**Authors:** Christine L. Miller, Tsute Chen, Ping Chen, Kai P. Leung

**Affiliations:** a Dental and Craniofacial Trauma Research and Tissue Regeneration, U.S. Army Institute of Surgical Research, JBSA Fort Sam Houston, Texas, USA; b The Forsyth Institute, Cambridge, Massachusetts, USA

## Abstract

Infection with *Pseudomonas aeruginosa* leads to impairment of healing and many deaths in severe burn patients. The phenotypic diversity of *P. aeruginosa* strains makes it difficult to define a therapeutic strategy. Here we report the genome sequence of a highly virulent strain of *P. aeruginosa*, VA-134, isolated from a burn patient.

## GENOME ANNOUNCEMENT

*Pseudomonas aeruginosa* is a Gram-negative bacterium and common burn wound pathogen (1–4). Factors leading to poor outcomes from burn patients include the invasive nature and virulence of the *P. aeruginosa* infecting strain and burn severity such as increased depth and coverage. *P. aeruginosa*’s virulence can be attributed to its metabolic versatility, adherence to and biofilm formation on the host, and the ability to evade and combat the host’s immune response (5). *P. aeruginosa* secretes toxic compounds and degradative enzymes (e.g., hemolysins, leukocidins, elastase, LasA protease, phospholipase C, exotoxin A, exoenzyme S, rhamnolipid, hydrogen cyanide [HCN], and pyocyanin) that contribute to pathogenesis (6, 7). The intrinsic and acquired biocide-resistance of *P. aeruginosa* strain VA-134 further hinder many treatment processes (8).

Studies using third-degree burn models in rats and burn wounds covering 20% (9, 10) or 30% (11) of the surface area, showed that *P. aeruginosa* strain VA-134, originally isolated from the urine of a burn victim, was the most virulent compared to strains isolated from other burn patients. Burned animals infected with VA-134 had the highest rates of mortality (9–11). Additionally, VA-134 demonstrated the greatest invasive potential leading to septicemia (9–11) and was less susceptible to phagocytosis and killing by granulocytes compared to other strains (11).

To determine the mechanisms underlying the highly virulent nature of the VA-134 isolate, the genome was sequenced. *De novo* genomic sequencing service was provided by BGI Tech Solutions Co., Ltd. (Cambridge, MA, USA) using a hybrid sequencing approach on two sequencing platforms—the Illumina HiSeq 4000 and PacBio RS II systems This approach generated a high-quality close-circular final assembly of 6,400,418-bp in size. The sequence was annotated by the NCBI Prokaryotic Genomes Automatic Annotation Pipeline (PGAP) (12), which generated the following: 5,854 genes, 5,725 coding sequences (CDS), 52 pseudogenes, 3 clustered regularly interspaced short palindromic repeat (CRISPR) arrays, 12 rRNAs, 64 tRNAs, and 1 noncoding RNA (nc-RNA). Bacterial culture is available from author K.P.L.

### Nucleotide sequence accession number.

This genome sequence was deposited in GenBank under the accession number CP013245.
